# Exploring decision-making performance in young adults with mental health disorders: a comparative study using the Cambridge gambling task

**DOI:** 10.1017/S0033291724000746

**Published:** 2024-07

**Authors:** R. Effah, K. Ioannidis, J.E. Grant, S.R. Chamberlain

**Affiliations:** 1 Department of Psychiatry, Faculty of Medicine, University of Southampton, Southampton, UK; 2 Southern Health NHS Foundation Trust, Southampton, UK; 3 Department of Psychiatry & Behavioral Neuroscience, University of Chicago, Pritzker School of Medicine, Chicago, IL, USA

**Keywords:** psychiatry, gambling, decision-making, cognition, impulsivity, risk, addiction, reward processing

## Abstract

Decision-making deficits, assessed cognitively, are often associated with mental health symptoms, however, this relationship is not fully understood. This paper explores the relationship between mental health disorders and decision-making, using the Cambridge Gambling Task (CGT). Our study investigated how decision-making varied across 20 different mental health conditions compared to controls in a sample of 572 young adults from the Minneapolis and Chicago metropolitan areas, using a computerized laboratory-based task. Almost all mental health conditions were associated with at least mild (i.e. at least small effect size) impairment in all three studied parameters of the CGT (risk adjustment, quality of decision-making and overall proportion of bet). Notably, binge eating disorder had the largest cognitive impairment and gambling disorder had moderate impairment. Post-traumatic stress disorder (PTSD) was associated with impaired decision-making while obsessive–compulsive disorder (OCD) and depression showed moderate impairment. Additionally, half of the disorders assessed had moderate to large impairment in risk adjustment.These findings suggest that mental health conditions may have a more complex cognitive profile than previously thought, and a better understanding of these impairments may aid in risk assessment and targeted clinical interventions. This study underscores the need for further research to determine the causal pathways between mental health conditions and cognition, as well as to better understand the day-to-day impact of such deficits.

## Introduction

Cognition can be broadly defined as the mental action or process of acquiring knowledge and understanding through thought, experience, and the senses. There are dissociable cognitive functions that have been implicated across a range of mental health conditions. A wide range of mental health disorders are associated with poor working memory, impaired decision-making, and attentional impairments (Gould, 2010; Grace, 2016; Murphy et al., 2001; Pironti et al., 2014; Rogers et al., 1999a).

The profile of cognitive impairment is important for understanding the neurobiological changes that may underpin these conditions, however pragmatically they are vital in understanding how pathologies manifest themselves in the day-to-day activities of individuals. Abnormalities of decision-making have been researched in mental health conditions. For example, a stark example of this relationship is in gambling disorder (as defined in the Diagnostic and Statistical Manual Version 5 [DSM-5] [American Psychiatric Association, 2013]): affected individuals repeatedly gamble, and make unwise decisions, despite negative consequences. Meta-analyses and systematic reviews have shown the differences in aspects of decision-making in gambling disorder documenting increased risk taking, impulsivity, and impaired judgment (Grant, Chamberlain, Schreiber, Odlaug, & Kim, 2011; Ioannidis, Hook, Wickham, Grant, & Chamberlain, 2019; Kertzman, Lidogoster, Aizer, Kotler, & Dannon, 2011; Kräplin et al., 2014). These changes may not be as apparent in other conditions. A better understanding of how cognition is affected in mental health pathologies may assist in the assessment of possible risk, as impaired cognition may lead to adverse outcomes for themselves or those around them.

Risk taking and impaired decision-making can be objectively quantified using the Cambridge Gambling Task (CGT) (Brand, Labudda, & Markowitsch, 2006; Rogers et al., 1999b; Yazdi et al., 2019), which is part of the Cambridge Neuropsychological Test Automated Battery (CANTAB) (Sahakian et al., 1988). The CGT has proven sensitive to impaired decision-making in groups of people with gambling disorder, *v.* groups of controls, with gamblers having a higher tendency to seek risk and prefer more immediate rewards (Kräplin et al., 2014). These changes are not specific only to gambling and can be potentially seen in other conditions. For example, use of the CGT in depressed patients have shown altered cognition with impaired reward processing of individuals (Halahakoon et al., 2020; Yazdi et al., 2019). Other studies have also shown decision-making changes in anxiety and ADHD (Murphy et al., 2001; Sørensen et al., 2017; Tolomeo, Matthews, Steele, & Baldacchino, 2018).

Furthermore, studies have employed the CGT to understand the neural substrates involved in decision-making (Clark, Cools, & Robbins, 2004). For example, in patients with cortical lesions, damage to the ventromedial prefrontal cortex affected betting (irrespective of the odds of winning) on the CGT, whereas insula cortex lesions were associated with problems adjusting bets as a function of the odds of winning (Clark et al., 2008; Clark, Manes, Antoun, Sahakian, & Robbins, 2003). Both types of brain lesion were associated with significant abnormalities in probability judgment. Continued research on the relationship between mental health disorders and cognition may lead to a greater understanding of the neurocircuitry that is implicated in the manifestation of these conditions.

Importantly, the relative profiles of decision-making abnormalities across these and other mental health disorders have not been well-characterized. Therefore, our study used the CGT in a large cohort with a rich profile of mental health psychopathology, allowing comparison to be made in how decision-making varies across diseases. The aim of the study was to explore the profile of decision-making performance (as indexed by the CGT) across a range of mental health disorders compared to people without the given disorder of interest (hereafter referred to as controls). We hypothesized that the decision-making impairments will be present across different mental health conditions compared to controls and this would be most significant in individuals affected by gambling disorder.

## Methods

### Sample

572 young adults (aged 18–29 years) were enrolled from general community settings using community advertisements in the Minneapolis and Chicago metropolitan areas were enrolled. Participants must have gambled at least five times in the past year (i.e. a proxy for some level of impulsive behavior) and be able to be interviewed in person to be included. Exclusion criteria for this study were hearing or vision problems that made performing cognitive tasks difficult, and an inability to understand and consent to the study – as determined by the study team following interview of the participants. Comorbidities were permitted in the clinical groups, for example if a given individual had both depression and anxiety, they were included in the clinical data for both the ‘depression’ computation and the ‘anxiety’ computation. Participants were recruited via media advertisements. Each participant received a $50 gift card to an online store as compensation. The Institutional Review Board of the University of Chicago approved the study and the consent statement. After receiving a complete description of the study, participants provided written informed consent. The authors assert that all procedures contributing to this work comply with the ethical standards of the relevant national and institutional committees on human experimentation and with the Helsinki Declaration of 1975, as revised in 2008.

### Assessments

Demographic variables, including age, biological sex at birth, self-reported gender, self-reported racial-ethnic identity, and highest level of education completed, were recorded for all participants. Subjects received an in-person psychiatric evaluation from a member of the research team trained in the administration of all the instruments employed, these were:

- The Mini International Neuropsychiatric Inventory (Sheehan et al., 1998). This was used to screen for depression, generalized anxiety disorder, post-traumatic stress disorder, panic disorder, alcohol use disorder, schizophrenia, obsessive–compulsive disorder, and substance use disorder.
- The Minnesota Impulsive Disorders Interview which screens for compulsive buying, kleptomania, trichotillomania, skin picking disorder, pyromania, intermittent explosive disorder, compulsive sexual behavior, and binge eating disorder (Chamberlain & Grant, 2018; Grant, 2008).
- ADHD World Health Organization Screening Tool Part A (ASRS v1.1) (Kessler et al., 2005), which screens for putative Adult ADHD diagnosis. For the ADHD definition, in keeping with standard recommendations (Kessler et al., 2005), endorsement of at least four of six ADHD symptoms on the ASRS Part A was deemed indicative of this disorder.
- The Structured Clinical Interview for Gambling Disorder (SCI-GD) (Grant, Steinberg, Kim, Rounsaville, & Potenza, 2004), which screens for a diagnosis of gambling disorder alongside the severity.

**Figure 1.**
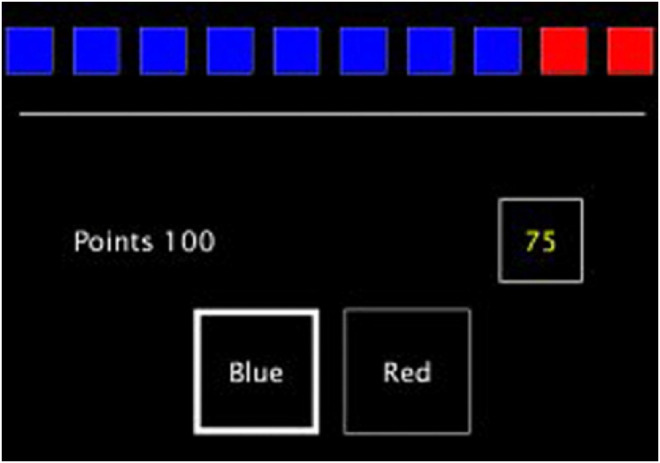
Image of the CGT © Copyright 2018 Cambridge Cognition Limited. All rights reserved.

The CGT from the CANTAB (Sandberg, 2011) was used to assess cognition of participants. The CGT was chosen as it is computerized, can fractionate distinct aspects of decision-making, and has received extensive investigations into its neurobiological underpinnings. In this assessment task ten blue and/or red boxes are presented in varying ratios (e.g. 7:3. 6:4) on a touch-sensitive computer screen. Participants then decide whether the yellow token is hidden in a red or blue box, staking a proportion of their cumulative points on their choice being correct. The proportions shown are 5, 25, 50, 75 or 95% randomly displayed in either descending or ascending order. If their choice was correct the stake is added to their total accrued points, or subtracted (i.e. lost) if incorrect. Participants are told that they should try and gain as many points as possible. Thus, the task explores different aspects of decision-making under controlled laboratory conditions (Fig. 1).

Three decision-making parameters were then recorded: the overall proportion of the bet, risk adjustment and the quality of decision-making. The overall proportion of the bet was the average proportion of points that was staked across the whole of the task. Risk adjustment was the degree to which a subject changed their risk taking in response to the ratios of red to blue boxes on each trial. Consistent riskier betting would lead to a lower risk adjustment score. A high risk adjustment score indicated a tendency to bet more in the rounds with better odds (Deakin, Aitken, Robbins, & Sahakian, 2004). Lastly, the quality of decision-making was the mean proportion of rounds where the most probable color (i.e. logical color choice) was chosen.

### Data analysis

Only psychiatric disorders endorsed by at least 1% (5 or more) of participants were included in the data analysis. To assess the degree of impairment as quantified using the CGT, results were calculated into relative *z*-scores (effect size) *v.* controls. For each disorder of interest, controls comprised everyone in the total sample who did not have that disorder. For example, the control group for depression consisted of all participants that did not have depression as assessed by our instruments. Individuals that had comorbidities were included in the clinical data of all assessed conditions. For instance, an individual with anxiety and depression was included in the *Z*-score computation for both anxiety and depression. The effect sizes were interpreted per convention using Cohen's D criteria, with 0.2 being regarded as small/mild, 0.5 being moderate, and lastly 0.8 being large. Scores represented the proportion of participants either below or above the average of the control group (Fig. 1).

## Results

The sample consisted of 572 participants, 65.7% were female and 34.3% male. The average age was 22.3 years old, with the majority (73.6%) having college education or higher. 72.1% were white Caucasian, 14.6% were African-American, and 6.3% were Asian. The remaining 7% consisted of Latino/Hispanic, Middle Eastern, Native American and mixed race. Table 1 shows the number of participants with each disorder.

**Table 1.** Number of participants with each mental health disorder

Mental health disorder	Number of participants with disorder
Any MINI	209
Depression	13
Panic disorder	7
Agoraphobia	24
Social anxiety disorder	24
OCD	12
PTSD	6
Alcohol dependence	80
Alcohol abuse	74
Substance dependence	46
Substance abuse	41
Bulimia nervosa	10
Generalized anxiety disorder	24
Antisocial personality disorder	31
ADHD	75
Intermittent explosive disorder	10
Pathological gambling	92
Compulsive sexual behavior disorder	14
Compulsive buying disorder	23
Binge-eating disorder	7

**Figure 2.**
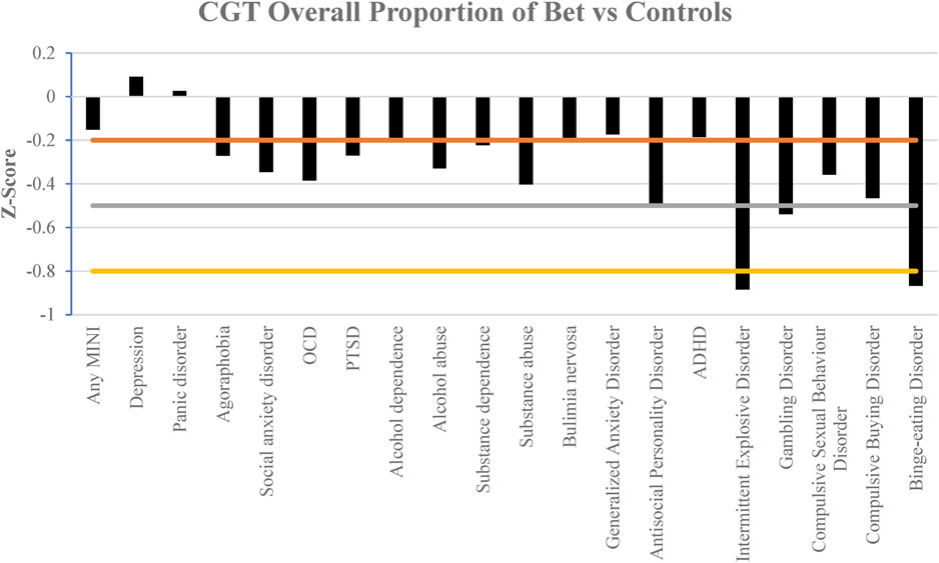
Comparison of the effect size in the overall proportion of bet staked in the CGT in different mental health disorders compared against controls. Horizontal lines indicate magnitude of impairment, mild (−0.2), moderate (−0.5) and large (−0.8 and less). A more negative *z*-score indicates larger bets *v*. controls.

**Figure 3.**
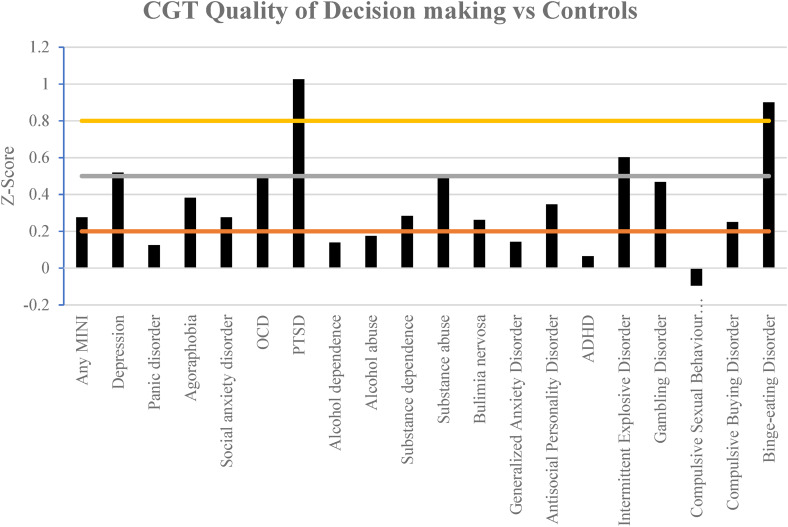
Comparison of the effect size in the quality of decision-making assessed via the CGT in different mental health disorders compared against controls. Horizontal lines indicate the magnitude of impairment, mild (0.2), moderate (0.5) and large (0.8 and greater). A more positive *z*-score indicates greater relative impairment *v.* controls.

**Figure 4.**
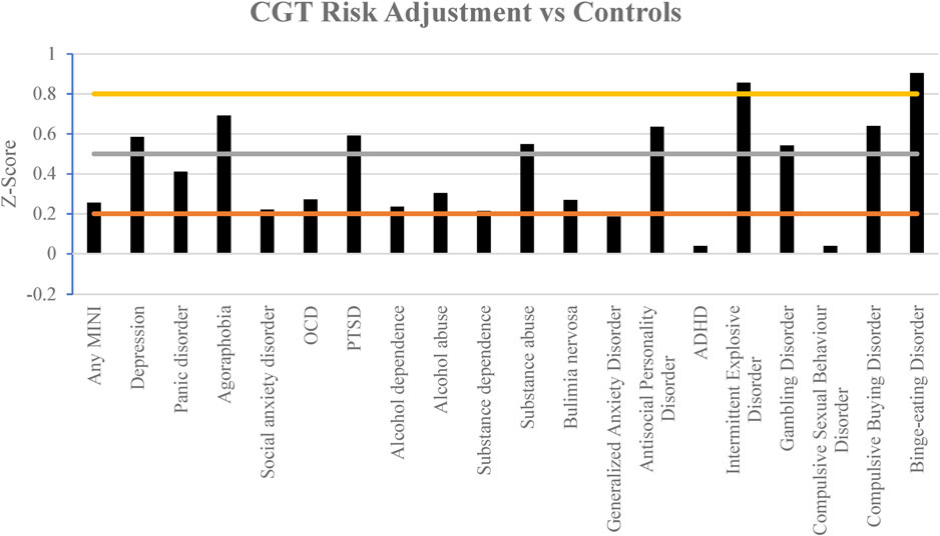
Comparison of the effect size in the adjustment of risk assessed via the CGT in different mental health disorders compared against controls. Horizontal lines indicate the magnitude of impairment, mild (0.2), moderate (0.5) and large (0.8 and greater). A more positive *z*-score indicates greater relative impairment *v.* controls.

Figures 2–4 illustrate the results of the three different parameters as assessed via the CGT. Almost all mental health disorders were associated with at least a mild impairment in all parameters, as compared to respective control groups of individuals without the given condition.

Interestingly gambling disorder was associated with moderate impairment consistently in all three parameters, whereas binge eating disorder had large impairment throughout. Participants with intermittent explosive disorder also had a large tendency to bet larger proportions of their points and exhibited large impairment in risk adjustment.

Participants with post-traumatic stress disorder (PTSD) had greater impairment in the quality of decision-making, whilst participants with major depressive disorder and obsessive–compulsive disorder (OCD) exhibited moderately impaired quality of decision-making (Fig. 3).

Participants' ability to manage risk and adapt was moderately impaired in half of the mental health disorders assessed. In contrast only ADHD and compulsive sexual behavior disorder failed to show at least a mild impairment on this domain (Fig. 4).

## Discussion

This study is one of the first to report on the relative profiles of decision-making impairments across a broad range of mental disorders, using a laboratory-based paradigm. The key findings were that almost all disorders were linked to at least mild (i.e. small effect size) impairment in one or more decision-making measures. Some disorders were more profoundly affected than others, as described in detail below.

These results importantly highlight that decision-making was impaired – in relative terms – across different conditions, and contrary to our expectation while gambling disorder was linked to deficits, some other disorders had more profound impairments. Notably, binge eating disorder had larger cognitive impairment in comparison to all other psychiatric disorders, whereas in contrast gambling disorder had a moderate impairment throughout. The fact that decision-making was particularly impaired in binge-eating is interesting in light of a recent systematic review and meta-analysis of the available literature (Colton, Wilson, Chong, & Verdejo-Garcia, 2023) – which found a range of impairments, across tasks and measures, relating to aspects of decision-making in binge-eating disorder. The authors suggested that the deficits could arise from a combination of processes, such as: difficulty forming stable preferences in situations involving ambiguous or complex outcomes; attentional response disinhibition; inflexibility; and difficulties using moment-by-moment feedback to optimize decisions (Colton et al., 2023). Thus, these processes (or a combination of them) may have contributed to the particularly pronounced deficits seen in the current study, though future work would be needed to confirm this.

Those with intermittent explosive disorder were also the only participants that had a large effect size tendency to make larger bets, with poorer risk adjustment. As one would expect, those with gambling disorder also bet more which is consistent with the known psychological profile of the disorder (Ioannidis et al., 2019).

Participants with PTSD had large impairment in their quality of decision-making which was an interesting result. This may be due to a reduced expectation of a positive outcome as seen in a similar study assessing reward processing in PTSD (Hopper et al., 2008; May & Wisco, 2020). Therefore, this may have led to participants believing the majority color was not as favorable due to a perception that regardless of their actions the rewards would be minimal (i.e. an ‘assumption of defeat’).

Most importantly, half of the mental health disorders assessed in this study showed a moderate to large impairment in risk adjustment, manifesting as a relative inability to flexibly adjust gambling behavior as a function of risk. However, it is unclear whether this is due to a tendency to engage in risky behavior. Two different strains of thought may be implicated in this result. Either the individual had a psychiatric disorder that caused them to be more risk averse, less pursuant in reward, and thus more rigid in their thinking e.g. PTSD, depression (Halahakoon et al., 2020; May & Wisco, 2020). Or alternatively the individual had a condition that is more risk prone such as gambling disorder (Kräplin et al., 2014). Therefore, they would be less likely to alter their risky behavior in pursuit of higher gains because their overall profile of approach was to take higher risks.

These results suggest that mental health conditions not conventionally studied/conceptualized in terms of cognition may nonetheless have complex psychological profiles that include a degree of relative decision-making impairment. A better understanding of such impairment may allow for clinical treatment to better assess the risk that could be involved with these disorders. A holistic appreciation of the psychological profile may allow clinicians to better understand behavior and the subsequent choices patients may make and work towards better outcomes. For example, cognitive training is being explored as a candidate intervention for gambling disorder (Luquiens, Miranda, Benyamina, Carré, & Aubin, 2019). Because some degree of deficit (in relative terms) was found across disorders, impaired decision-making may not be specific to any disorder, but could be viewed as a trans-diagnostic treatment target, and possibly an indication of vulnerability. Relatedly, the overlapping deficits may reflect overlapping comorbidities across disorders.

## Limitations

Despite examining decision-making profiles across disorders, it is important to consider a number of limitations. Firstly, it is important to note that the CGT examines aspects of decision-making and is not designed to fully capture ‘impulsivity’ – especially in terms of disinhibition, which is better measured using tasks such as stop-signal paradigms. As a naturalistic study comparing people with *v.* without each mental health condition of interest, cognitive findings could have been contributed to by other variables (such as comorbidities, or demographic differences between groups). Some of the disorders studied had relatively small sample sizes, and it will be important to attempt to replicate findings using larger samples in future. The sample's average age was 22.4 years old, being majority female, which may reduce the generalizability of the data (Rolison, Hanoch, Wood, & Liu, 2014). Some research has shown that age and gender can influence CGT performance to some degree (Deakin et al., 2004; Rolison et al., 2014). Future work should address the influence of age and gender, and other potential confounding variables (e.g. comorbidities), but this would require a larger sample than was available here. The participants in this sample were non-treatment seeking which could reduce the applicability of the findings to people presenting in clinical settings. In addition, we were unable to control for co-morbidities or the use of medications or drugs that affect cognition due to the small sample size and the nature of the study. As comorbidities are common in mental health conditions it is likely that some participants had more than one disorder. Another limitation is that we focused on effect sizes, due to the variable and in some cases relatively small sample sizes in particular groups, rather than conducting formal statistical *p* values tests. Some of the disorders studied had relatively small sample sizes, and it will be important to attempt to replicate findings using larger samples in future. Lastly, as a cross sectional study, these results cannot determine causality between mental health disorders and cognition. Addressing this issue would require a very large-scale longitudinal study with inclusion of detailed screening for such a broad range of mental disorders, including gambling disorder (which is often overlooked).

## Conclusion

In conclusion, our study presented the relative decision-making profiles of people with a range of mental health disorders, using a computerized laboratory-based task. The findings highlight the need to consider decision-making not only in conditions conventionally likely to be linked to impairments, such as gambling disorder, but also other conditions such as PTSD and binge-eating disorder. If these findings generalize to people with these conditions, the relative cognitive deficits may constitute trans-diagnostic targets for treatment interventions, or indicate the need to adapt existing treatments in order to help address these additional difficulties.
